# The clinical and cost-effectiveness of elective primary total knee replacement with PAtellar Resurfacing compared to selective patellar resurfacing: a pragmatic multicentre randomized controlled Trial (PART): the clinical and cost-effectiveness of elective primary total knee replacement

**DOI:** 10.1302/2633-1462.56.BJO-2023-0154

**Published:** 2024-06-03

**Authors:** Adam Boon, Elizabeth Barnett, Lucy Culliford, Rebecca Evans, Jessica Frost, Zastra Hansen-Kaku, William Hollingworth, Emma Johnson, Andrew Judge, Elsa M. R. Marques, Andrew Metcalfe, Patricia Navvuga, Michael J. Petrie, Katie Pike, Vikki Wylde, Michael R. Whitehouse, Ashley W. Blom, Gulraj S. Matharu

**Affiliations:** 1 Bristol Trials Centre, University of Bristol Faculty of Health Sciences, Bristol, UK; 2 Southmead Hospital,, North Bristol NHS Trust, Westbury-on-Trym, Bristol, UK; 3 Population Health Sciences, University of Bristol, Bristol, UK; 4 Musculoskeletal Research Unit, Bristol Medical School, University of Bristol, Bristol, UK; 5 National Institute for Health Research Bristol Biomedical Research Centre, University of Bristol, Bristol, UK; 6 Warwick Clinical Trials Unit, Warwick Medical School, University of Warwick, Coventry, UK; 7 Sheffield Teaching Hospitals NHS Foundation Trust, Sheffield, UK; 8 University of Sheffield, Sheffield, UK

**Keywords:** Total knee arthroplasty, patellar resurfacing, selective resurfacing, patient-reported outcomes, randomized controlled trial

## Abstract

**Aims:**

During total knee replacement (TKR), surgeons can choose whether or not to resurface the patella, with advantages and disadvantages of each approach. Recently, the National Institute for Health and Care Excellence (NICE) recommended always resurfacing the patella, rather than never doing so. NICE found insufficient evidence on selective resurfacing (surgeon’s decision based on intraoperative findings and symptoms) to make recommendations. If effective, selective resurfacing could result in optimal individualized patient care. This protocol describes a randomized controlled trial to evaluate the clinical and cost-effectiveness of primary TKR with always patellar resurfacing compared to selective patellar resurfacing.

**Methods:**

The PAtellar Resurfacing Trial (PART) is a patient- and assessor-blinded multicentre, pragmatic parallel two-arm randomized superiority trial of adults undergoing elective primary TKR for primary osteoarthritis at NHS hospitals in England, with an embedded internal pilot phase (ISRCTN 33276681). Participants will be randomly allocated intraoperatively on a 1:1 basis (stratified by centre and implant type (cruciate-retaining vs cruciate-sacrificing)) to always resurface or selectively resurface the patella, once the surgeon has confirmed sufficient patellar thickness for resurfacing and that constrained implants are not required. The primary analysis will compare the Oxford Knee Score (OKS) one year after surgery. Secondary outcomes include patient-reported outcome measures at three months, six months, and one year (Knee injury and Osteoarthritis Outcome Score, OKS, EuroQol five-dimension five-level questionnaire, patient satisfaction, postoperative complications, need for further surgery, resource use, and costs). Cost-effectiveness will be measured for the lifetime of the patient. Overall, 530 patients will be recruited to obtain 90% power to detect a four-point difference in OKS between the groups one year after surgery, assuming up to 40% resurfacing in the selective group.

**Conclusion:**

The trial findings will provide evidence about the clinical and cost-effectiveness of always patellar resurfacing compared to selective patellar resurfacing. This will inform future NICE guidelines on primary TKR and the role of selective patellar resurfacing.

Cite this article: *Bone Jt Open* 2024;5(6):464–478.

## Trial summary

Controlling pain and improving mobility in the long term after knee replacement surgery has been highlighted as a research priority by patients. Knee replacement is common (109,000/year in the UK), and is performed to help patients with pain from disabling arthritis. There are two ways to carry out this surgery. In about two-thirds of knee replacements, the kneecap (patella) is unaltered during the operation. In the remaining third of operations, the surgeon attaches a separate artificial implant to the back of the kneecap, which may help reduce further wear or pain. This is known as resurfacing the kneecap. Resurfacing is an extra step in the operation which takes time and sometimes causes problems later on. Not resurfacing can cause long-term knee pain, and further surgery may be needed, resulting in risks for patients and expense to the NHS. Recent national guidelines compared resurfacing the kneecap in all patients with never resurfacing the kneecap, and concluded that in the long term, resurfacing in all cases was better than never resurfacing.

However, many surgeons make an individual choice about whether to resurface the kneecap, based on factors such as pain and the condition of the kneecap. We call this selective resurfacing. The National Institute for Health and Care Excellence (NICE) highlighted a need for research about whether selective resurfacing is better than always resurfacing during knee replacement.

This study will compare whether it is better for patients if surgeons resurface every patient’s kneecap during knee replacement, or if surgeons only resurface the kneecap when they believe it will lead to a better outcome.

## Introduction

Total knee replacement (TKR) is a clinically and cost-effective surgical procedure for treating patients with severe arthritis.^1^ In the UK, over 100,000 primary TKRs are performed a year, costing the NHS £550 million annually.^2,3^ TKR volume continues to rise each year.^4^

During TKR, the bottom of the femur and the top of the tibia are replaced with implants. Intraoperatively, surgeons have the option to perform a further procedure known as patellar resurfacing. This involves removing the under-surface of the patella and attaching a plastic prosthesis, known as a patella button. Alternatively, when not resurfaced, the native patella cartilage articulates with the femoral implant of the TKR. The National Joint Registry (NJR) for England, Wales, Northern Ireland, the Isle of Man, and Guernsey shows that out of 1,100,000 primary TKRs performed since 2003, the majority (62%) have not had the patella resurfaced.^5^

The decision whether or not to perform patellar resurfacing in TKR is controversial. Some surgeons always resurface the patella while others never do so. There are potential advantages and disadvantages of each. Proponents of patellar resurfacing claim that if not resurfaced, 25% of patients develop chronic anterior knee pain with poor outcomes and dissatisfaction.^6^ This adversely affects patient-reported outcome measures (PROMs) and can lead to further surgery (secondary patellar resurfacing) in 7% of patients, with associated risks and NHS costs.^6^ Secondary patellar resurfacing surgery may not correct the problem once it occurs, with poor patient satisfaction in up to 64% of patients who undergo it and low rates of clinical improvement.^7,8^ Opponents of patellar resurfacing propose that resurfacing is an additional step in the operation which is not needed, given studies show that PROMs are similar between patients undergoing patellar resurfacing compared with those not having patellar resurfacing.^9-11^ Performing patellar resurfacing extends surgical time by up to ten minutes, increasing costs beyond that of the implant, and increases the risk of intraoperative complications, such as patella fracture and patella tendon injury.^6^

There is evidence that always patellar resurfacing (compared with never resurfacing) results in lower revision rates within ten years of primary TKR surgery, and is cost-effective given fewer patients need additional surgery in the long term.^11,12^ In the NICE guidance published in June 2020, a strategy of always patellar resurfacing was recommended over never resurfacing based on the available evidence.^3^ A third option called selective patellar resurfacing (individualized intraoperative decision made by the surgeon based on the state of the patellar surface and the patient’s symptoms) was considered by NICE; however, no evidence was found, so a research recommendation was made for a future trial comparing always to selective patellar resurfacing. Selective patellar resurfacing could be a more effective strategy than always resurfacing, as it potentially preserves the benefits of both approaches.

Selective patellar resurfacing can be considered a patient-specific treatment approach, in which the surgeon decides on a case-by case basis whether or not to resurface the patella based on their experience and an interoperative patient-specific assessment. Selective patellar resurfacing may potentially improve PROMs compared with always resurfacing by only resurfacing the subgroup of patellae, which are judged at higher risk of causing pain now and in the future if they were not resurfaced; by not resurfacing those patients where the surgeon thinks that resurfacing is not needed or high-risk, disadvantages and complications of resurfacing are prevented, such as overstuffing, maltracking, patellar fracture, and implant loosening, all of which lead to worse PROMs. There are also potential cost savings from decreased theatre time and patellar implant costs.

There is no high-quality evidence comparing always versus selective patellar resurfacing.^13^ It is important to know which strategy is best for patients and the NHS.^3^ The 2020 NICE joint replacement guidelines identified, ‘In adults having elective knee replacement, what is the clinical and cost effectiveness of TKR with patellar resurfacing compared with selective resurfacing?’ as a research priority.^3^

## Aims and objectives

The overall aim of this study is to evaluate the clinical and cost-effectiveness of always resurfacing the patella compared to selective resurfacing in elective primary TKR.

Specific objectives of the trial are to: 1) estimate the difference between groups in the mean Oxford Knee Score (OKS) at one year postoperatively;^14,15^ 2) estimate the difference between groups with regard to a range of secondary outcomes, including knee-related PROMs (Knee injury and Osteoarthritis Outcome Score (KOOS)^16^ and OKS), health-related quality of life (EuroQol five-dimension five-level questionnaire (EQ-5D-5L)),^17^ complications, further surgery, and resource use up to one year postoperatively; 3) analyze the cost-effectiveness (cost per quality-adjusted life year; QALY) of always patellar resurfacing compared to selective patellar resurfacing at one year postoperatively; and 4) model longer-term outcomes using routinely collected data (e.g. need for further knee surgery using the NJR and NHS Hospital Episode Statistics (HES)) and extrapolate the cost-effectiveness results beyond the trial using historical NJR/HES data to estimate revision rates and costs in an economic Markov model.

## Plan of investigation

### Trial design

**Fig. 1:**
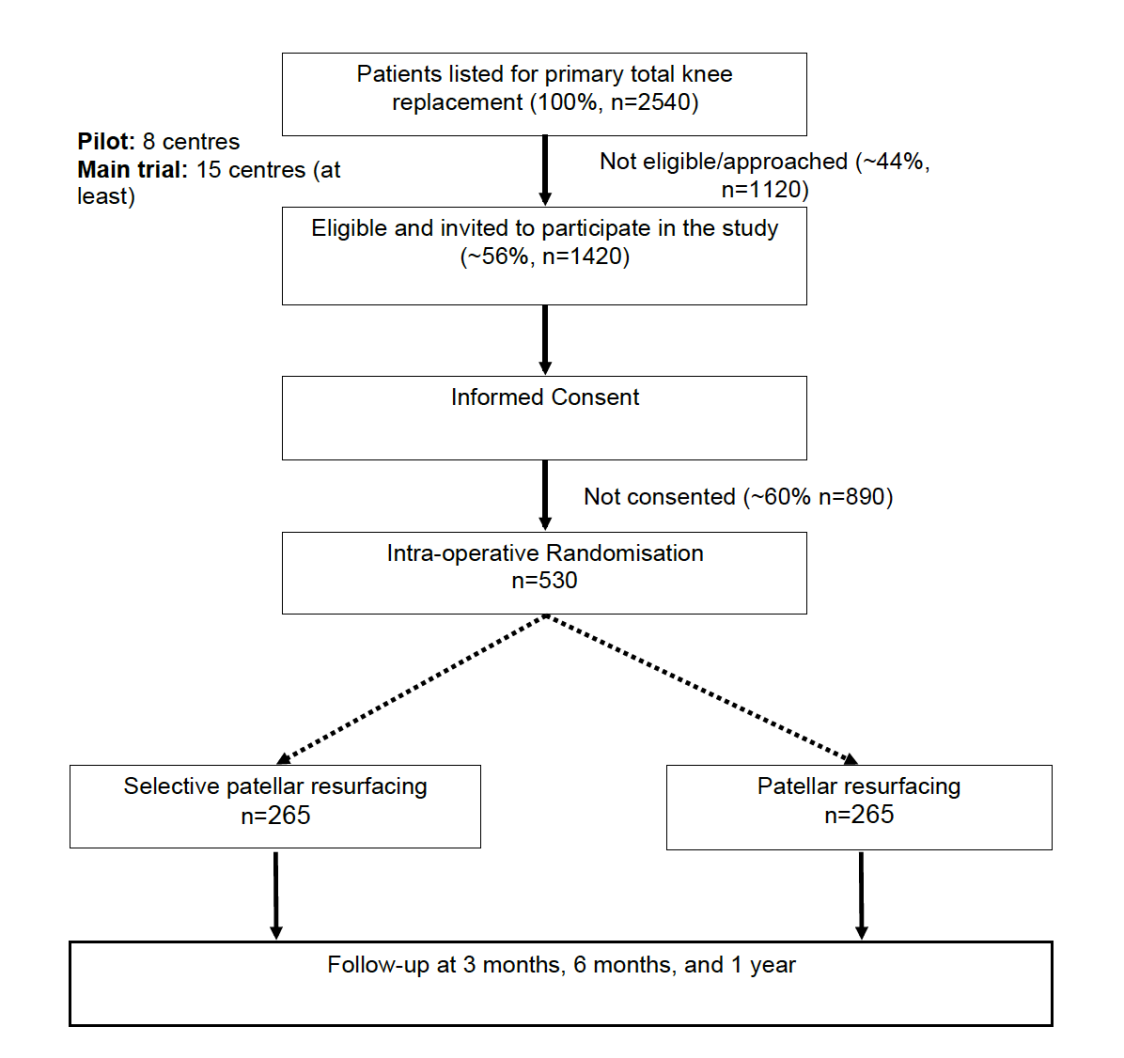
Trial overview.

The trial is a multicentre, pragmatic parallel two-group superiority randomized controlled trial (RCT), in which patients, clinical care teams (except for staff involved in the theatre itself, such as the surgeon), and members of the research team responsible for data collection will be blinded to allocation (Figure 1).

### Internal pilot

The trial set-up will take six months, and internal pilot will take place in at least eight sites for eight months to assess trial procedures, and to maximize recruitment and surgeon adherence. Progression is contingent on meeting defined criteria.

### Main trial

During the main trial phase, the study will aim to open at least seven more sites (total at least 15 recruiting sites) using optimized methods from the internal pilot. The main trial phase will run over 16 months. All participants will be followed up for 12 months.

### Setting

Patients will be recruited from secondary and tertiary care NHS hospitals in England.

### Key design features to minimize bias

**1) Selection bias/allocation bias** (systematic differences between baseline characteristics of the groups that are compared): this bias is ruled out by concealed randomization, which will be performed intraoperatively. The allocation will not be revealed until sufficient information to uniquely identify the participant and establish eligibility has been entered into the trial database.

**2) Performance bias** (systematic differences between groups in the care that is provided, or in exposure to factors other than the interventions of interest). This bias will be minimized by: defining the interventions and the standard protocols for all other aspects of care during the study; defining procedures for follow-up; blinding the clinical care team not directly involved in the surgery, those responsible for data collection, and study participants; and monitoring adherence to the protocol.

The patient information leaflet (PIL) and the process of obtaining informed consent will describe the uncertainty about the clinical benefit of patellar resurfacing over selectively resurfacing. Therefore, in the event of inadvertent unblinding of a participant, he or she should not have a strong expectation that any one method should lead to a more favourable result.

**3) Attrition bias** (systematic differences between groups in withdrawals from a study). This bias will be minimized by: using established Bristol Trials Centre (BTC) methods to maximize the quality and completeness of the data and minimize non-adherence (e.g. regular monitoring of data, detailed querying of data inbuilt into the study database, offering alternative methods for participating in follow-up (e.g. postal, online, or telephone)); implementing measures to promote adherence to random allocations. Any instances of non-adherence will be fully documented and reviewed at study meetings, and an action plan for maximizing compliance drawn up as appropriate.

Data will be analyzed by intention-to-treat (ITT) (i.e. according to the treatment allocation, irrespective of future management and events), and every effort will be made to include all randomized patients. Participants will be blinded and so a differential dropout rate across the two groups should not be seen.

**4) Reporting bias** Reporting bias will be minimized by pre-specifying study outcomes, publishing the trial protocol, and following a detailed analysis plan which will be prepared in advance of any comparative analyses of the study data. Participants and staff responsible for collecting data will be blinded to reduce reporting bias.

### Trial population

Adults listed for elective primary TKR for osteoarthritis (OA) at secondary and tertiary care NHS hospitals in England.

### Inclusion criteria

Participants may enter the study if ALL of the following apply: adults (aged ≥ 18 years); elective primary TKR for primary OA; and resident of England (English postcode) and/or surgery in an English NHS hospital.

### Exclusion criteria

Participant may not enter the study if ANY of the following apply: revision TKR, unicompartmental knee replacement, or primary elective TKR with: need for constrained implants (e.g. constrained condylar or hinge); isolated patellofemoral OA; history of septic arthritis; diagnosis other than primary OA; intraoperative patellar thickness insufficient for safe patellar resurfacing as determined by the treating surgeon (patellar thickness will be recorded in the case report forms for monitoring purposes); patient is unable/unwilling to adhere to trial procedures; and/or patient is unable to provide written informed consent. Participating in another study that may affect the outcomes of this trial or that does not permit co-enrolment in another study, or where co-enrolment would be burdensome to the patient, will also lead to exclusion from this trial. This will be assessed on a case-by-case basis by the local principal investigator (PI), in consultation with the co-chief investigators (CIs).

### Trial interventions

Treatments will be delivered under the care of a consultant orthopaedic surgeon. As a pragmatic trial, there will be no restrictions in anaesthetic (general or regional anaesthesia), antibiotic and perioperative medication use, or surgical technique (including tourniquet use, approach, fat pad resection, implants used, alignment, and soft-tissue balancing). These aspects of care are at the discretion of the treating surgeon and anaesthetist. Key details about the intervention and other aspects of care will be collected in the case report forms (CRFs).

Both trial interventions are stable, and it is not anticipated that there will be significant change to the interventions during the trial. All study surgeons will deliver the study treatment.

### Surgical procedure: all patients

The knee joint will be exposed using the surgeon’s routine approach. Patella thickness will be measured with a calliper as is routine practice, and recorded in the CRFs. Once trial eligibility is confirmed by the surgeon, the patient will be randomized intraoperatively to either patellar resurfacing or selective patellar resurfacing.

### Surgical comparator: patellar resurfacing

All patients in this group will undergo patellar resurfacing, according to the surgeons’ preferred technique and implants. Typically, this additional procedure takes five to ten minutes longer to perform compared with not performing patellar resurfacing.

### Surgical intervention: selective patellar resurfacing

Surgeons will use their individual judgement and experience to decide whether or not to perform patellar resurfacing. The surgeon will make this decision based on the patients’ preoperative clinical features and symptoms, and their intraoperative assessment. Patellar resurfacing will be undertaken as per routine practice.

### Site and surgeon eligibility

The techniques used in this study are stable clinical interventions that are in frequent and widespread use across the NHS. The skills required to perform the interventions are held by all knee surgeons performing primary TKR.

## Primary and secondary outcomes

Our proposed outcome measures align with the recently published OMERACT core outcome set for knee OA research.^18^

### Primary outcome

The primary outcome is the OKS one year after TKR surgery. It consists of 12 items, with a total score ranging from 0 (worst knee pain/function) to 48 (no pain or functional problem).^14,15^

### Secondary outcomes

Data will be collected on the following secondary outcomes:

**Knee pain and function:** OKS at three and six months; OKS pain and function subscales at three months, six months, and one year; and KOOS at three months, six months, and one year. KOOS is a 42-item PROM validated for use in TKR patients.^19^ The KOOS assesses knee-specific pain, symptoms, activities of daily living, sport and recreation function, and quality of life, with a total from 0 to 100 (worst to best). The minimal clinically important difference (MCID) is eight to ten points.^19^ The KOOS is an extension of the Western Ontario and McMaster Universities Osteoarthritis Index (WOMAC), which is commonly used in the USA for assessing outcomes following treatment for arthritis. The KOOS can thus be used to calculate a WOMAC score, which would aid comparison with previous studies reporting WOMAC.^19^

**Health-related quality of life:** EQ-5D-5L at three months, six months, and one year, which is a validated, generalized, and standardized self-reported instrument assessing health-related quality of life.^17^ It consists of a visual analogue scale measuring self-rated health, and a health status instrument of five domains related to quality of life. This instrument will be used to derive QALYs.

**Patient satisfaction:** four-item satisfaction questionnaire.

**Complications:** within one year of surgery and to include venous thromboembolism (deep vein thrombosis and/or pulmonary embolism), bleeding, infection, fracture, non-fracture extensor mechanism failure (tendon or quadriceps rupture), further surgery revision (includes secondary patellar resurfacing, and debridement and implant retention), intensive care unit periods, and death.

**Length of surgery:** from skin incision to end of wound closure, in minutes.

**Postoperative hospital stay:** as required by standard of care in days.

**Resource use:** for the one year of trial follow-up, data will be extracted from hospital records by the site research nurses, including outpatient appointments and readmissions required to treat complications related to TKR surgery, and also from participant questionnaires at three months, six months, and one year collecting further health and care use specifically related to knee problems. The latter questionnaires will include questions about primary care, physiotherapy, and other community-based healthcare related to the knee post-randomization, as well as collecting data on return to work/usual activities, social care, and informal care requirements.

**Further surgery of the patella:** any further surgical intervention performed on the patella.

### Sample size calculation

The standard deviation (SD) for the OKS after primary TKR is ten points.^1,11,15^ The OKS Minimally Clinical Important Difference (MCID) is four points.^20^ Assuming: 1) a correlation of 0.5 between the pre-surgery and post-surgery OKS, and 0.7 between repeated post-surgery scores (conservative estimates), which provides an efficiency gain equivalent to reducing the SD from 10 to 7.4; 2) that up to 5% may not undergo resurfacing in the always resurfacing group and that up to 40% will undergo resurfacing in the selective resurfacing group; and 3) a 5% two-sided statistical significance level and 90% power, we require 530 patients in total (265 per group) allowing for an estimated 10% loss to follow-up.^11^

## Trial methods

### Participant recruitment

All patients listed for primary TKR for OA will be invited to participate. Potential trial participants will be identified by local site clinical teams. Prior to screening, patients will be seen in arthroplasty or specialist knee clinics in consultation with a knee surgeon. During the clinic appointment, the clinician will review the patient’s information, including radiographs, and once the patient is confirmed as requiring a primary TKR for symptomatic OA, they will be listed for surgery. Patients may be on the waiting list for up to 12 to 18 months (waiting times will vary between centres). Patients may be informed of the study at the clinic, but due to long waiting times, it is anticipated that patients may be identified from existing surgery waiting lists and approached closer to their surgery date, for example at a preoperative assessment clinic or consent clinic.

There will be a three-stage screening process. The initial stage of screening will take place once the patient is identified on the surgery waiting list or from a virtual or face-to-face clinic consultation. This will involve assessment of the eligibility criteria, such as the diagnosis of primary OA. If none of the exclusion criteria have been met, the patient will be approached and given a patient information leaflet (PIL) at this stage, either in person at a clinic, or they will be sent the PIL in the post or via email by a member from the research team. If patients are sent a PIL in the post or via email, a member of the local site research team may have a telephone consultation or video call to explain the study and answer any questions. The PIL will include contact details for the research team in case the patient has any questions.

Following this, at two to eight weeks before surgery, the patient will attend a routine preoperative assessment or other clinic (this will vary across sites). The surgeon will confirm the patient’s preoperative eligibility and a member of the research team will receive consent (if the patient decides to participate). All individuals receiving informed consent will be good clinical practice (GCP) trained. During the consultation potential participants will be fully appraised of the potential risks, benefits, and burdens of the study. They will also be informed that if the patella is found to be too thin for resurfacing intraoperatively, they will not be eligible for the study and will receive standard care. If a site is not able to take consent at a preoperative clinic, providing the patient has had time to consider the study and ask any questions, written consent can be taken on the day of surgery by a member of the research team. The patient will keep a copy of the consent form, the research team will file the original consent form in the investigator site file (ISF), and a copy will be stored in the patient’s medical records. Details of all patients approached for the trial and reason(s) for non-participation (e.g. reason for being ineligible or patient refusal) will be documented.

The final stage of screening will occur in theatre when the patient’s patella thickness is measured, and it is confirmed that there is no requirement for constrained implants. At this point, patient eligibility for the study can be fully confirmed. The patient will therefore be randomized in theatre.

### Study information pack and consent provision

All potential patients will receive an invitation letter and PIL, approved by the Health Research Authority (HRA)/NHS research ethics committee (REC), describing the study as part of a study information pack. These documents may be given to the potential patient in person or sent via post or via email. The study information pack, if sent by post or email, may also include the PART patient consent form and baseline questionnaire for completion before surgery if the patient consents to join the trial. Whether the questionnaire is sent and completed before attending the hospital for surgery, or is completed when the patient attends the hospital, will depend on the local patient pathway. A second baseline questionnaire will be completed if the participant’s initial questionnaire was completed more than six months before their surgery date.

We will ask patients to consent to have their data linked to NJR data to allow for long-term follow-up data to be collected at five and ten years post-randomization. Consent will be obtained either face-to-face at a clinic appointment, remotely by telephone/video call, or electronically using a purpose-designed electronic database called REDCap (Vanderbilt University, USA). The consent process will be described in detail in the study manual. Participants who consent via video call or telephone will be guided through the process of completing the consent form by the local research team. Participants will be asked to return their signed consent form by scanning or taking a photograph of the form(s) and emailing the form(s), posting the form(s) to the research team, bringing the form(s) to their next hospital visit, or submitting the online e-consent form.

On receiving the consent form(s), the research team will check for errors, countersign, and date. Photocopies of the consent form(s) will be made and the research team will ensure that the participant is given a copy of their countersigned consent form(s) at a hospital visit or is sent copies by post or email as preferred. The countersigned consent form will be retained at the study site, and a copy will be filed in the medical notes. Details of all participants approached for the study and reason(s) for non-participation (e.g. reason for being ineligible or participant refusal) will be documented. Eligibility will be confirmed by a clinician prior to randomization.

## Description of randomization and code breaking

### Randomization

Randomization will be carried out intraoperatively, once patient eligibility is confirmed by the surgeon (i.e. that a highly constrained TKR is not needed and the patella is thick enough to resurface). Consent and baseline assessment will be completed prior to surgery. Randomization will be performed by a member of the research team not involved in data collection or participant follow-up using a secure internet-based system to ensure allocation concealment. If a member of the research team were unavailable in theatre, a member of the clinical team could perform the randomization. Participants will be allocated in a 1:1 ratio to either patellar resurfacing or selective patellar resurfacing. The allocation will be computer-generated, stratified by centre and implant type (cruciate-retaining or -sacrificing), and blocked using blocks of varying size. Surgeon treatment intent (i.e. resurface or not if randomized to the selective group) will be collected immediately prior to randomization to assess adherence to the planned strategy. Any barriers to randomization once eligibility has been confirmed will be explored in the internal pilot.

### Manual randomization

Instructions on how to perform a manual randomization will be provided to the local research team should the online randomization system fail.

### Blinding

Patients, their clinical care team (except for staff directly involved in the surgery), and researchers responsible for follow-up will not be informed of the allocation. Other than radiologically, it is not possible to know if the patella has been resurfaced, so we do not expect participants to be unblinded. Researchers responsible for data collection and follow-up will not randomize patients, and will not be in the operating theatre or have access to any knee radiographs. We will assess the success of blinding by asking the patient and all outcome assessors which treatment they think was received using the Bang Blinding Index.^21^ Initially, this will be done during the pilot phase to assess the responses and determine if it is useful to continue in the main trial. Both staff and patients will be asked. We will use blinded operation notes, as we have done successfully in previous National Institute for Health and Care Research (NIHR)-funded trials START:REACTS (NIHR16/61/18),^22^ RACER (NIHR128768),^23^ and SISMIC (NIHR127849),^24^ and ask surgeons to not show participants their radiographs during follow-up to reduce the risk of unblinding.

### Unblinding

We do not anticipate unblinding will be requested on clinical grounds (e.g. to treat a complication). The management of any serious adverse event (SAE) (e.g. infection or bleeding) would not be altered by knowledge of the allocation. However, should it be necessary, review of any postoperative radiograph will allow immediate and easy unblinding. Unblinding rates will be monitored throughout the trial by the study team and by the independent data monitoring and safety committee (DMSC). Participants will be made aware before entering the study that they will not be told which treatment they will receive until the end of the trial.

## Research procedures

### Patient-reported outcomes

Patient-reported outcome questionnaires (OKS, KOOS, and EQ-5D-5L) will be administered prior to randomization as part of this study. Consented patients will be asked to complete a baseline questionnaire. Final eligibility for the trial will be confirmed during surgery; participants deemed ineligible at this point will not be randomized and no further data collection will occur. Participants confirmed to be eligible for study inclusion after intraoperative assessment will complete questionnaires at three months, six months, and one year post-randomization. The PROM questionnaires will be collected by post, telephone, or online, as per patient preference.

### Treatment adherence

The planned surgical strategy (i.e. resurface or not) will be captured before randomization. Problems with adherence are expected to be low given that randomization will take place after trial eligibility is confirmed intraoperatively. Data on the factors that influence a surgeon’s decision-making on selective resurfacing as identified in our national survey will be collected for all participants, to allow adherence to be monitored to assess any bias and/or lack of equipoise among surgeons, which will be assessed formally during the internal pilot.

### Rehabilitation procedure: all participants

There will be no restrictions on the rehabilitation and physiotherapy protocol after surgery. Patients will have clinical follow-up as per usual care for each centre, which is typically between six and 12 weeks and at one year following TKR. No additional research-specific visits are required for this trial. When routine follow-up appointments align with the time period for collecting outcomes for the trial, outcome data will be collected at these appointments.

### Duration of treatment period

The duration of the treatment commences when the patient enters the operating theatre and concludes when the patient leaves the operating theatre after their surgery. Performing patella resurfacing is usually an additional five to ten minutes of operating time.

### Frequency and duration of follow-up

Follow-up will utilize a range of methods to meet with patients (e.g. face-to-face, via phone, or video call). Questionnaires will be administered at approximately three months, six months, and one year post-randomization for information on knee function, HRQoL, complications, and resource use, as well as on the effectiveness of blinding (at three and 12 months only). Guidance on how, and when, to send these questionnaires will be provided in the study manual provided to sites.

Participants will attend the site for routine follow-up at approximately three months, and one year depending on local arrangements (trust-/hospital-specific follow-up schedule). Data will be collected at these visits, with alternative arrangements for participants who do not attend (e.g. postal or online data collection, telephone follow-up at mutually agreed times), or for those where the hospital does not have a scheduled appointment at the timepoint when the study questionnaires require completion.

All questionnaires will be administered by a researcher at each participating centre in person, by post, or online. Participating centres will be responsible for collecting these from patients and entering them into the study database. A reminder will be sent approximately two weeks after the initial contact if no reply has been received, followed by a telephone call to allow completion of the questionnaire with a researcher. Further follow-up may continue for up to ten years subject to further funding, and patients will be asked whether or not they will consent to longer-term follow-up before entering the study.

### Likely rate of loss to follow-up

Until discharge from hospital, the only losses to follow-up will be due to participant withdrawal; these losses are expected to be very few. We expect loss to follow-up at one year post-randomization to be no more than 10% on the basis of previous studies.^11^ This potential loss to follow-up has been accounted for when estimating our sample size.

### Data collection

The data collection is outlined in Table I.

**Table I.** Data collection.

Data item	Baseline	Intraoperative	Discharge	Post-randomization		
				3 months	6 months	12 months
Demography	*✓*					
Relevant medical history	*✓*					
Comorbidities	*✓*					
Operative details		*✓*				
Confirmed eligibility		*✓*				
Length of hospital stay			✓			
Bang Blinding Index				*✓*		*✓*
OKS	*✓*			*✓*	*✓*	*✓*
KOOS	*✓*			*✓*	*✓*	*✓*
HRQoL (EQ-5D-5L)	*✓*			*✓*	*✓*	*✓*
Postoperative complications			*✓*	*✓*	*✓*	*✓*
SAEs, including readmissions*			*✓*	*✓*	*✓*	*✓*
Resource use		*✓*	*✓*	*✓*	*✓*	*✓*

* Serious adverse events (SAEs) will be subject to expedited reporting to the Sponsor up to three months post-randomization. SAEs collected for later time points will not be subject to expedited reporting.

EQ-5D-5L, EuroQol five-dimension five-level questionnaire; HRQoL, health-related quality of life; KOOS, Knee injury and Osteoarthritis Outcome Score; OKS, Oxford Knee Score; SAE, serious adverse event.

Each patient will be assigned a unique study number. All data recorded on paper relating to the participant will be located in CRF folders, which will be stored securely at individual sites. Staff with authorization to make changes to the study records, including the study database, will be listed on the study delegation log maintained at each centre. The baseline data will be collected after consent. Consenting patients will be seen by an authorized member of the local research team (as specified in the delegation log) who will answer any questions, confirm the patient’s eligibility, and receive written informed consent if the patient decides to participate. Patients who choose to consent using electronic consent methods (e-consent) will verbally provide their email address to the local research team to receive a link to the electronic consent form.

Data collection will include the following elements: a screening log of all patients identified (prospectively, or retrospectively from current procedure waiting lists) who are awaiting elective primary TKR for primary osteoarthritis will be invited to participate; patients approached and assessed against the eligibility criteria and, if ineligible, reasons for ineligibility collected; consent information collected prior to randomization in all participating patients; baseline information (e.g. sociodemographic, history, planned operation, and response to health/comorbidities/work status questionnaires) collected in all participating patients; data relating to the participant’s surgery and hospital stay collected in all participating patients; data on health status, activity, knee function, productivity (collected via questionnaires), adverse events, and resource use collected at three months, six months, and one year post-randomization for all participating patients; and mortality.

To minimize bias, outcome measures are defined as far as possible on the basis of objective criteria. All personnel carrying out outcome assessment will be blinded; this will minimize detection bias.

### Source data

The primary data source will be the participant’s medical records, alongside the data collection forms for the study. The completed patient questionnaires will be the primary data source for information on the patients’ health, knee function, and comorbidities. These data will be supplemented by Hospital Episode Statistics and NJR data to facilitate long-term economic modelling beyond the trial at five and ten years post-randomization.

### Discontinuation/withdrawal of participants

Each participant has the right to withdraw at any time. It is unlikely for this trial that there would be any reason for the investigator to withdraw the participant from their allocated treatment, unless subsequent to randomization a clinical reason for not performing the surgical procedure is discovered. There are no specific criteria for withdrawal. However, a clinician may withdraw a participant from treatment at any time if they feel it is in the participant’s best interests. In the unlikely event that a participant loses capacity during the study, they will be withdrawn.

All withdrawals, including reasons (where given), will be captured in the study database and reported. If a participant wishes to withdraw, data collected up until that point will be included in the analyses.

Passive data collection (e.g. from medical records, registry data, and routinely collected data) will continue, unless the participant expresses a wish for this to stop. This is explained in the PIL.

### Definition of end of trial

Active data collection will continue up to one year post-randomization. The patient’s active involvement in the trial will end at this point. Data collection for the whole trial will be complete when the final randomized participant has completed the one-year post-randomization assessments. The end of the trial will be when the database is closed and all the data queries have been answered.

## Trial management

North Bristol NHS Trust will act as the sponsor. The trial will be managed by the BTC. The BTC is built on the experience of the Bristol Clinical Trials and Evaluation Unit and the Bristol Randomized Trials Collaboration, both fully registered UK Clinical Research Collaboration (UKCRC) units. The BTC will prepare all the trial documentation and data collection forms, specify the randomization scheme, develop and maintain the study database, check data quality as the trial progresses, monitor recruitment, and carry out trial analyses in collaboration with the CIs.

### Day-to-day management

Appropriately qualified persons by training will be responsible for identifying potential trial participants, seeking informed participant consent, randomizing participants, collecting trial data, and ensuring the trial protocol is adhered to.

The core research team will meet approximately every four to six weeks to manage the trial and monitor progress. The core team are regular collaborators on a large number of different projects and in the case of the clinicians, work together in delivering elective patient care. There are well-established lines of communication, and such communication will be continuous throughout the life of the project rather than being constrained to formal meetings only, which will facilitate rapid response to any issues raised.

## Monitoring of sites

### Site initiation

Before the study commences, training session(s) will be organized by the BTC. These sessions will ensure that personnel involved fully understand the protocol, CRFs, and the practical procedures for the study. These sessions will either be completed face-to-face or via teleconference.

### Site monitoring

The BTC will carry out central monitoring and audit of compliance of centres/surgical specialties with the principles of GCP and data collection procedures. The study database will have extensive in-built validation, and the core research team and trial management group will review the completeness and consistency of the data throughout the trial. The BTC will not check CRFs against the data entered or against source data, unless there are good reasons to visit the site to complete a monitoring visit (e.g. the central monitoring highlights a problem or as requested by the sponsor).

## Trial steering committee and data monitoring and safety committee

### Trial steering committee

An independent trial steering committee (TSC) will be established to oversee the conduct of the study. It is anticipated that the TSC will comprise an independent chair and at least three additional independent members, including a statistician or methodologist, an orthopaedic knee surgeon, an experienced clinical researcher, and a patient/public involvement (PPI) representative. The PPI coordinator will support the patient/public representative if required. The TSC will develop terms of reference outlining their responsibilities and operational details. The TSC will meet before recruitment begins and regularly (at intervals to be agreed with the committee) during the course of the study. The TSC will formally review recruitment after ten months and make recommendations.

### Data monitoring and safety committee

An independent DMSC will be established to review safety data during the course of the study, and will review the assumptions underpinning the sample size calculation. The DMSC will develop a charter outlining their responsibilities and operational details. The DMSC will meet (jointly with the TSC) before the trial begins and regularly thereafter (at intervals to be agreed with the committee).

## Safety reporting

**Fig. 2:**
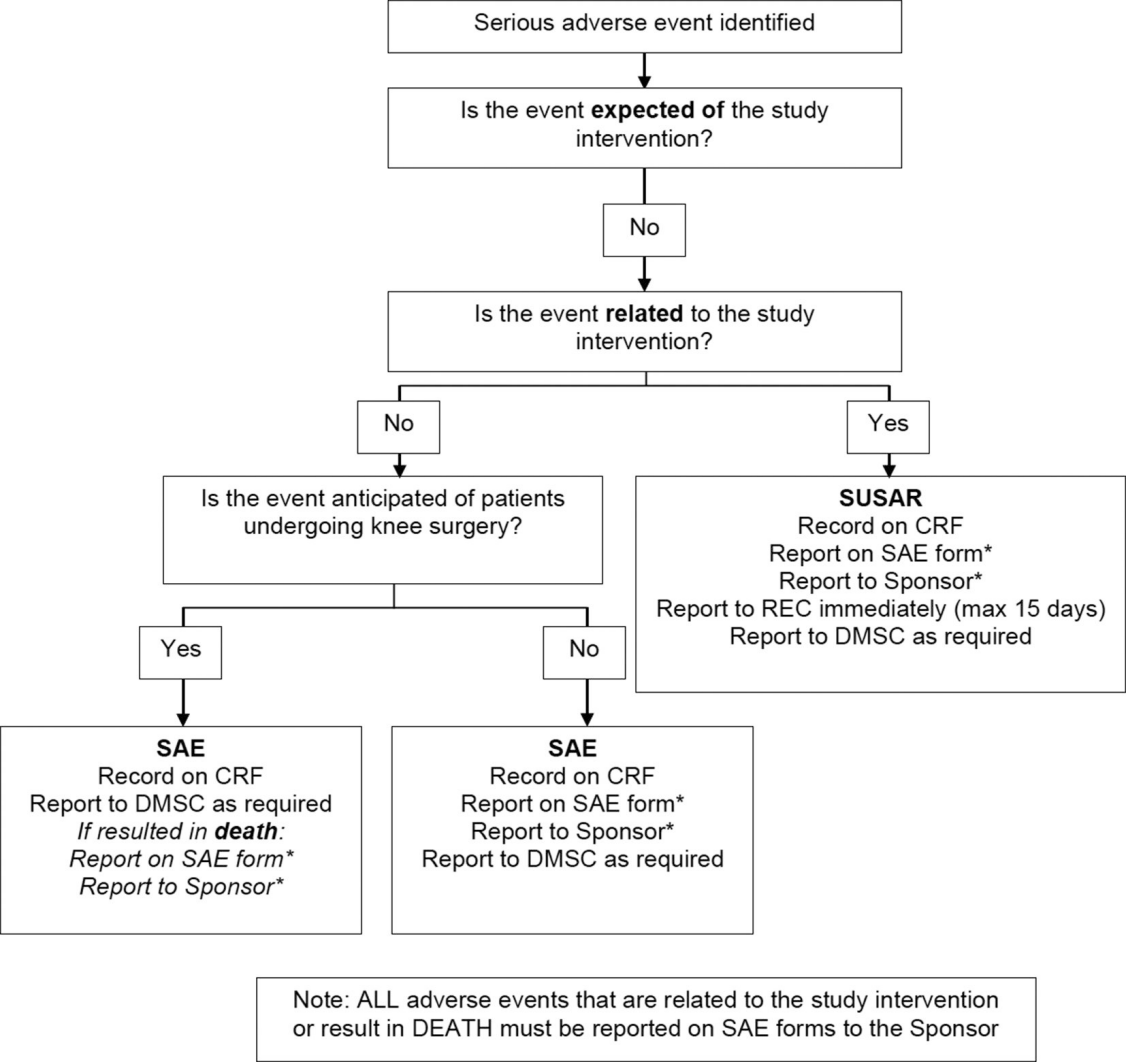
Serious adverse event (SAE) reporting flowchart. *SAEs will be subject to expedited reporting to the sponsor up to three months post-randomization, unless the SAE is related. Related SAEs will be subject to expedited reporting to the sponsor up to 12 months post-randomization. Beyond the three-month timepoint, aggregated reports will be provided to the sponsor. CRF, case report form; DMSC, data monitoring and safety committee; REC, Research Ethics Committee; SUSAR, suspected unexpected serious adverse reactions.

Serious and other adverse events will be recorded and reported in accordance with GCP guidelines and the sponsor’s standard operating procedue (SOP) (see Figure 2 and Table II for definitions).

**Table II.** Safety reporting definitions.

Term	Definition
Adverse event (AE)	An AE can be any unfavourable or unintended sign (including an abnormal laboratory finding), symptom, or disease temporarily associated with the research procedure, whether or not considered related. AEs require continuous assessment.
Adverse reaction (AR)	The distinguishing feature between an AR and AE is whether there is evidence to suggest there is a causal relationship between the event and the research procedure.
Serious adverse event (SAE)	Any untoward medical occurrence that: results in death is life-threatening requires inpatient hospitalization or prolongation of existing hospitalization results in persistent or significant disability/incapacity consists of a congenital anomaly or birth defect Other ‘important medical events’ may also be considered serious if they jeopardize the participant or require an intervention to prevent one of the above consequences. NOTE: The term ‘life-threatening’ in the definition of ‘serious’ refers to an event in which the participant was at risk of death at the time of the event; it does not refer to an event which hypothetically might have caused death if it were more severe.
Serious adverse reaction	Any SAE that is classed in nature as serious and there is evidence to suggest there is a causal relationship between the event and the research procedure, but where that event is expected.
Suspected unexpected serious adverse reaction	Any SAE that is classed in nature as serious and there is evidence to suggest there is a causal relationship between the event and the research procedure, but where that event is unexpected.

The BTC will report suspected unexpected serious adverse reactions (SUSARs) to regulatory authorities and copy all reports to the sponsor within the expected timeframes. Sites will report SAEs to the BTC within 24 hours of the study team becoming aware of the event. Events that are anticipated of surgery will not require expedited reporting to the sponsor unless they are deemed to be related to the intervention, otherwise all unexpected serious events will be reported to the sponsor.

Elective surgery during the follow-up period that was planned prior to recruitment to the trial will not be reported as an unexpected SAE. If the event is ongoing, there is no mandatory requirement regarding the frequency with which follow-up reports should be submitted. As a minimum, a report should be submitted when the event resolves/ends.

### Expected events of selective patellar resurfacing

There are no known expected events associated with the study intervention, selective resurfacing of the patella, as these risks are similar to the study comparator (always resurfacing), such as patella instability and fracture. A proportion of patients in this group will receive patella resurfacing (the study comparator, and also the standard of care recommended by NICE), and a proportion of patients in this group will not be resurfaced.

### Anticipated events of knee surgery

The following adverse events occur frequently in patients undergoing knee surgery, and have been highlighted as adverse events following TKR by experts/professional societies, and therefore will be considered anticipated:

- Swelling that meets the criteria of a serious event, or requires further surgical intervention (e.g. further arthroscopic or open surgery).
- Pain that meets the criteria of a serious event, or requires further surgical intervention (e.g. further arthroscopic or open surgery).
- Stiffness that meets the criteria of a serious event, or requires further surgical intervention (e.g. further arthroscopic or open surgery or a manipulation under anaesthetic).
- Infection as confirmed by positive microbiological samples from the operated knee or requiring washout or debridement for infection.
- Bleeding requiring washout in theatre.
- Scarring: excessive scarring leading to stiffness or another problem that requires further surgical intervention (e.g. further arthroscopic or open surgery or a manipulation under anaesthetic).
- Patella tendon injury, patella fracture, and/or non-fracture extensor mechanism failure or disruption.
- Nerve damage: leading to a persistent (more than two weeks) alteration in motor function of a peripheral nerve or sensory disturbance.
- Venous thromboembolism (deep vein thromosis/pulmonary embolism).
- Complications, which may or may not lead to further knee surgery, including wound complication, vascular injury, medial collateral ligament injury, instability, malalignment, fracture, patellofemoral dislocation, tibiofemoral dislocation, bearing surface wear, osteolysis, implant loosening, implant fracture/tibial insert dissociation, revision, and readmission.
- Further knee surgery not captured from the above reasons.

Data on these adverse events collected during the trial will be reported regularly to the trial DMSC and to the sponsor for review. If an anticipated event meets the criteria for seriousness (as outlined in Table II) and is deemed by the principal investigator (or delegated individual) to be possibly, probably or definitely related to the study intervention this event would be reported as a SUSAR.

### Period for recording serious adverse events

Data on adverse events will be collected from randomization to hospital discharge. All SAEs will be collected from consent up to 12 months post-randomization. All SAEs will be subject to expedited reporting to the sponsor up to three months post-randomization. Thereafter, related SAEs will be subject to expedited reporting to the sponsor up to 12 months post-randomization and all other SAEs will be reported to the sponsor in periodic aggregated reports.

## Statistical analyses

### Plan of analysis: primary and secondary outcomes

Primary analyses will be by ITT and will be directed by a pre-specified statistical analysis plan. Analyses will use data from all randomized patients. The primary outcome and continuous secondary outcomes measured at multiple timepoints will be analyzed using a mixed regression model, which will include an interaction between treatment and time, to allow the effect of the treatment strategy to be quantified for each postoperative timepoint. Binary outcomes will be analyzed using a generalized linear model; the risk difference and risk ratio will be reported. Length of surgery and length of hospital stay will be analyzed using generalized linear models. Model validity will be checked using standard methods; if a model is a poor fit, alternative models or transformations will be explored. Outcomes analyzed on a logarithmic scale will be transformed back to the original scale after analysis and results presented as geometrical mean ratios. Analyses will be adjusted for baseline scores where measured, and variables used to stratify the randomization will be fitted as random effects. Adverse events will be reported using the medical dictionary for regulatory activities classification system.^25^ Findings will be reported as effect sizes with 95% confidence intervals, and in accordance with the CONSORT reporting guidelines.

Participants will be asked to consent to have their data linked to NJR data to allow long-term follow-up and for the research team to carry out a supplementary check on the data.

Full details of statistical analyses will be pre-specified in a publicly available statistical analysis plan (SAP) in accordance with the guidelines for the content of SAPs in clinical trials.^26^

### Subgroup analyses

No subgroup analyses are planned.

### Frequency of analyses

The primary analysis will take place when follow-up is complete for all recruited patients (i.e. at one year post-randomization). No interim analysis of outcomes is planned. Safety data will be reported to the DMSC at a frequency agreed by the committee, together with any additional analyses the committee request.

### Criteria for the termination of the trial

Conditions that might lead the TSC to recommend stopping the trial early include: 1) failure to recruit sufficient patients or open sufficient sites to meet the target sample size within the proposed duration of the grant and refusal of the funder to extend the duration of recruitment; and 2) a failure to deliver the intervention as planned.

With regard to 1), our progression criteria are detailed in Table III. The pilot will recruit in eight centres over eight months. The pilot will monitor: recruitment rates (proportion of screened patients who are eligible, eligible patients consented and confirmed eligible at surgery); adherence to the allocated treatment/planned resurfacing strategy (to assess any bias/lack of equipoise among surgeons); and rates of resurfacing in both groups.

**Table III.** Progression criteria.

Criteria	Target	Red	Amber	Green
Centres open to recruitment, n	8	< 6	6 to 7	8
Recruitment target, n	42	< 33	33 to 41	42
Randomization rate/centre/month open	2.8	< 2.3	2.3 to 2.7	2.8
Adherence to the allocated treatment/planned resurfacing strategy, %	100	< 80	80 to 99	100
Percentage resurfaced in the selective group, %	≤ 40	> 50	41 to 50	≤ 40

Recruitment to similar studies by this research team has been feasible, so major barriers to recruitment are unlikely.^1,11,18^ It is accepted that recruitment typically starts slowly and increases over time as the trial gets established, and that there is some variability from one month to the next (e.g. recruitment is typically lower over Christmas and in the summer holiday period than at other times of year). Strategies will be developed in collaboration with our PPI partners to tackle any barriers identified by collecting reasons for non-participation. The trial team will prepare a report for the TSC to consider and make a recommendation to the NIHR-HTA.

With regard to 2) (failure to deliver the intervention as planned), we will monitor adherence to the protocol throughout the trial and investigate all cases of non-adherence. Patellar resurfacing rates in the two groups will be reviewed by DMSC for the trial, who will advise whether there is sufficient separation between groups for the full trial to be feasible. If the trial proceeds from the internal pilot to the main trial, patients from the internal pilot will be included in the final analysis. We will prepare a report for the TSC to consider, and we will propose halting the trial if the reasons for non-adherence cannot be addressed satisfactorily.

If all criteria are green, we will proceed to a full trial with the same protocol; if one or more criteria are amber, we will propose adaptions to address the shortfall; if one or more criteria are red, we will discuss with the TSC and the NIHR whether the full trial is feasible. In addition to monitoring recruitment and adherence, the DMSC will monitor safety outcomes. The DMSC may recommend stopping the trial if the accrued data suggest that the trial is unsafe for one or both groups of participants.

### Economic analyses

A within-trial economic evaluation will be conducted from an NHS perspective based on ITT for all randomized patients, following NICE guidelines.^27^ This will estimate the differences in the costs and health benefits between the two strategies in a cost-utility analysis. QALYs will be estimated based on mortality and EQ-5D-5L scores at three months, six months, and one year, adjusted for baseline scores, using the area under the curve approach.^28^ The NICE-recommended scoring algorithm at the time of the analysis will be applied to the responses to generate QALYs.^29^

The incremental cost of the initial surgical admission between the always and selective patellar resurfacing arms will be micro-costed based on data collected in the CRF on theatre and recovery room time, implants used, critical care, and hospital length of stay. Initial admission (measured on the CRF) and subsequent resource use (measured in patient questionnaires) will be valued using national unit costs for health and social care when available,^30,31^ or from hospital procurement systems (e.g. implant costs). Missing cost and QALY data will be estimated using multiple imputation methods where appropriate.^32^ Cost-effectiveness will be expressed in terms of incremental net monetary benefit statistics and 95% confidence intervals using NICE-recommended thresholds. The probability of always resurfacing being cost-effective will be depicted in a cost-effectiveness acceptability curve.^33^ Secondary analyses will explore the impact of treatment strategy on informal and social care costs and return to work/usual activities. Full details of economic analyses, including the long-term model, will be pre-specified in a publicly available health economics analysis plan.

## Ethical considerations

### Review by an NHS Research Ethics Committee

The research will be performed subject to a favourable opinion from an NHS REC and HRA, including any provisions of site-specific assessment (SSA), and local site capacity and capability confirmation. Ethics review of the protocol for the trial and other trial-related essential documents (e.g. PIL and consent form) has been carried out by a UK NHS REC (Welsh REC 2). Any subsequent amendments to these documents will be submitted to the REC and HRA for approval prior to implementation.

### Risks and anticipated benefits

Potential benefits of taking part in the study include that if either of the treatment arms is found to be superior, of which there is no current robust evidence, then patients allocated to that arm would receive a superior treatment. Conversely, those allocated to the other arm would not receive this benefit.

Participation in research studies may offer benefit to patients in terms of outcomes experienced for their treatments.

The risks, side-effects, and potential complications associated with participation in the study are the same between the control and intervention being used; as such, it is not anticipated that participation in the study would represent an increased risk for participants. Patients deemed to be eligible for inclusion will have end-stage OA that requires treatment with primary TKR as defined by NICE; therefore, the surgical treatment rate would not be increased for participants. Potential adverse effects of the types of surgery being used in this study include infection, bleeding, pain, stiffness, swelling, deep vein thrombosis, pulmonary embolism, scarring, numbness, and reoperation.

The conduct of this study will allow us to determine which of the treatments is the most clinically and cost-effective. As such, this study will allow us to make evidence-based recommendations for the treatment of this patient population.

### Informing potential study participants

Information about possible benefits and risks of participation will be described in the PIL.

### Obtaining informed consent from participants

All participants will be required to give written informed consent. This process, including the information about the trial given to patients in advance of recruitment, is described previously. The PI or delegate will be responsible for the consent process.

### Co-enrolment

Co-enrolment with another study will be considered on a case-by-case basis. Generally, co-enrolment will be allowed if the intervention is not expected to influence the primary outcome, it is permitted by the other study, and if participation in both studies does not present an excess burden to the participant.

## Research governance

This study will be conducted in accordance with GCP guidelines and UK Policy Framework for Health and Social Care Research.

### Sponsor approval

Any amendments to the trial documents must be approved by the sponsor prior to submission to the REC/HRA.

### Confirmation of capacity and capability

Confirmation of capacity and capability from each NHS trust is required prior to the start of the study at that site. Any amendments to the study documents approved by the REC and the HRA will be submitted to the study sites, as required by the HRA.

### Investigators’ responsibilities

Investigators will be required to ensure that local research approvals have been obtained and that any contractual agreements required have been signed off by all parties before recruiting any participant. Investigators will be required to ensure compliance to the protocol and study manual, and completion of the CRFs. Investigators will be required to allow access to study documentation or source data on request for monitoring visits and audits performed by the sponsor or the BTC or any regulatory authorities.

Investigators will be required to read, acknowledge, and inform their trial team of any amendments to the trial documents approved the REC/HRA that they receive and ensure that the changes are complied with.

### Monitoring by sponsor

The study will be monitored and audited in accordance with the sponsor’s policy, which is consistent with the UK Policy Framework for Health and Social Care Research and the Medicines for Human Use (Clinical Trials) Regulations 2004.^34^ All study-related documents will be made available on request for monitoring and audit by the sponsor (or the BTC if they have been delegated to monitor), the relevant REC/HRA, and for inspection by the MHRA or other licensing bodies.

### Indemnity

This is an NHS-sponsored research study. For NHS-sponsored research, HSG (96)48 reference no. 2 refers. If there is negligent harm during the clinical trial when the NHS body owes a duty of care to the person harmed, NHS Indemnity covers NHS staff, medical academic staff with honorary contracts, and those conducting the trial. NHS Indemnity does not offer no-fault compensation and is unable to agree in advance to pay compensation for non-negligent harm.

## Data protection and participant confidentiality

### Data protection

Data will be collected and retained in accordance with the UK Data Protection Act 2018.

### Data handling

Full details will be provided in the data management plan, which will also define how personal identifiable and non-identifiable patient information is used in the study. Data will be entered into a purpose-designed database hosted on the University of Bristol network. Database access will be password-controlled and restricted to PART trial staff at the participating site and the coordinating centre.

Any information capable of identifying individuals will be held on a secure University of Bristol server. PART trial staff at the coordinating centre will have access to this identifiable information. If required, this information can be securely shared with participating sites who will contact potential participants, for the purposes of the study. No personally identifiable data will be held on the study database.

The processing of participants’ personal data will be minimized by making use of a unique participant trial number on trial documents and the study database, with the exception of signed consent forms and the screening log. The database and randomization system will be designed to protect participant information in line with data protection legislation. Trial staff will ensure that the participant’s confidentiality is maintained through secure handling and storage of participant information at participating sites and in accordance with ethics approval.

Data will be entered promptly with data validation and cleaning to be carried out throughout the trial. The trial manual will cover database use, data validation, and data cleaning. The manual will be available and regularly maintained.

### Data storage

All study documentation will be retained in a secure location during the conduct of the study and for ten years after the end of the study, when all patient-identifiable paper records will be destroyed by confidential means. In compliance with the Medical Research Council (MRC)’s policy on data sharing, and with participant agreement, relevant metadata about the trial and the full dataset, but without any participant identifiers other than the unique participant identifier, will be held indefinitely (university server). These will be retained because of the potential for the raw data to be used subsequently for secondary research.

Archiving will be done as per the BTC SOPs in agreement with the sponsor. Sites will be expected to archive their own documents as per site agreements, and the BTC will archive the ISF and central coordinating centre documents for five years after the end of the study.

### Data sharing

Data will not be made available for sharing until after publication of the main results of the study. Thereafter, anonymized individual patient data will be made available for secondary research, conditional on assurance from the secondary researcher that the proposed use of the data is compliant with the MRC policy on data sharing regarding scientific quality, ethical requirements, and value for money. A minimum requirement with regard to scientific quality will be a publicly available pre-specified protocol describing the purpose, methods, and analysis of the secondary research (e.g. a protocol for a Cochrane systematic review).

## Dissemination of findings

The results of the study will be made publicly available within 12 months of the last patient’s last visit. The findings will be disseminated by usual academic channels, i.e. presentation at national and international meetings, as well as by peer-reviewed publications (including a full report to the NIHR-HTA programme) and through patient organizations and newsletters to patients, where available. Patients who state they would like to be updated on the results of the study will receive a summary of results at the end of the study.

**Take home message**

- The research question of the PAtellar Resurfacing Trial (PART) addresses a key area of discussion among surgeons: whether following National Institute for Health and Care Excellence guidance and resurfacing all patellae, or only resurfacing patellae when surgical judgement indicates it, is the best course of action.

## Data Availability

The data that support the findings for this study are available to other researchers from the corresponding author upon reasonable request.
